# Prognostic Assessment of Cervical Cancer Patients by Clinical Staging and Surgical-Pathological Factor: A Support Vector Machine-Based Approach

**DOI:** 10.3389/fonc.2020.01353

**Published:** 2020-08-05

**Authors:** Lin Xie, Ran Chu, Kai Wang, Xi Zhang, Jie Li, Zhe Zhao, Shu Yao, Zhiwen Wang, Taotao Dong, Xingsheng Yang, Xuantao Su, Xu Qiao, Kun Song, Beihua Kong

**Affiliations:** ^1^Department of Obstetrics and Gynecology, Qilu Hospital, Cheeloo College of Medicine, Shandong University, Jinan, China; ^2^Department of Obstetrics and Gynecology, Jining No.1 People's Hospital, Jining, China; ^3^School of Control Science and Engineering, Shandong University, Jinan, China; ^4^Gynecology Oncology Key Laboratory, Qilu Hospital, Shandong University, Jinan, China

**Keywords:** cervical cancer, clinical staging, surgical-pathological staging, Sedlis criteria, support vector machine

## Abstract

**Introduction:** The International Federation of Gynecology and Obstetrics (FIGO) staging system is considered the most powerful prognostic factor in patients with cervical cancer. In addition, other surgical-pathological risk factors have been demonstrated to have significance in predicting the prognosis of patients. Therefore, the purpose of this study was to investigate the effects of the FIGO staging system and surgical-pathological risk factors on the prognosis of cervical cancer patients.

**Methods:** A retrospective study was performed on patients diagnosed with cervical cancer at FIGO stage IB1–IIA2. Kaplan–Meier, Cox proportional hazards regression analysis and the support vector machine (SVM) algorithm were used to assess and validate the high-risk factors related to recurrence and death.

**Results:** A total of 647 patients were included. Kaplan-Meier analysis showed that five high-risk factors, including FIGO stage, status of pelvic lymph node, parametrial involvement, tumor size, and depth of cervical cancer, had a significant effect on the prognosis of patients. In multivariate analysis, pelvic lymph node metastasis (hazard ratio [HR] 2.415, 95% confidence interval [CI] 1.471–3.965), parametrial involvement (HR 2.740, 95% CI 1.092–6.872) and >2/3 depth of cervical invasion (HR 2.263, 95% CI 1.045–4.902) were three independent risk factors of disease-free survival. Pelvic lymph node metastasis (HR 3.855, 95% CI 2.125–6.991) and parametrial involvement (HR 3.871, 95% CI 1.375–10.900) were two independent risk factors for overall survival. When all five high-risk factors were assembled and used for classification prediction through SVM, it achieved the highest prediction accuracy of recurrence (accuracy = 69.1%). The highest prediction accuracy for survival was 94.3% when only using the two independent predictors (the pathological status of lymph nodes and parametrium involvement) by SVM classifiers. Among the 13 groups of intermediate-risk factor, the combination of tumor size, histology and grade of differentiation was more accurate in predicting prognosis than the intermediate-risk factors in the Sedlis criteria (recurrence: 86.8% vs. 60.0%; death: 92.0% vs. 71.6%).

**Conclusions:** The combination of FIGO stage and surgical-pathological risk factors can further enhance the prediction accuracy of the prognosis in patients with early-stage cervical cancer. Histology and grade of differentiation can further improve the prediction accuracy of intermediate-risk factors in the Sedlis criteria.

## Introduction

Cervical cancer is the second most common malignancy, and the third most common cause of cancer death worldwide in females (1). Because only one in five Chinese women reported having a screening test for cervical cancer, there is a substantial increase trend in China, which is in contrast to the decrease in cervical cancer incidence observed in developed countries (2, 3).

Clinicopathologic risk factors, such as pelvic lymph node metastasis, parametrial involvement, lymphovascular space invasion, tumor size, depth of cervical invasion and histology, have been identified to have prognostic significance in cervical cancer (4–9). Among these risk factors, deep stromal invasion, large tumor size, and lymphovascular space invasion are defined as intermediate-risk factors (10). Moreover, when these factors are combined, they increase the risk of postoperative recurrence by 15–20% (11). Although pathological factors can influence the prognosis of patients, clinical staging is still suggested by the International Federation of Gynecology and Obstetrics (FIGO) in cervical cancer (12). However, clinical staging of cervical cancer is based primarily on pelvic examination by a gynecologist before any therapy is performed. It is inherently inaccurate if the patient has pelvic inflammatory disease, endometriosis, or obesity (13). Cervical cancer staging entails individual subjective judgments.

Surgical-pathological staging was mentioned in recent studies and is viewed as an ideal method to determine the extent of the disease by histopathologic examination (13, 14). Moreover, in 2018, the FIGO Gynecologic Oncology Committee first allowed imaging and pathological findings to assign the clinical stage of cervical cancer, which demonstrated the importance of pathological factors in the assessment of prognosis (15). Therefore, in addition to the clinical staging of the FIGO system, more accurate methods to predict recurrence and survival are critical to adjuvant treatment in cervical cancer patients.

The aim of this study is to combine the FIGO staging system and surgical-pathological factors to explore their impact on the prognosis of patients with early-stage cervical cancer, and provide a reference for clinical precision treatment.

## Materials and Methods

### General Information

Patients who were diagnosed with early-stage cervical cancer (FIGO stage IB-IIA) and had been treated at Qilu Hospital of Shandong University between January 2005 and December 2016 were enrolled in our study. The Ethical Committee in Qilu Hospital of Shandong University approved this study (2018066) and provided a waiver for informed consent. Our study included patients who met the following criteria: (1) FIGO stage IB-IIA (2009 FIGO staging system) (16) and (2) underwent radical hysterectomy with pelvic lymphadenectomy. The exclusion criteria were as follows: (1) preoperative neoadjuvant chemotherapy or radiotherapy; (2) unusual histology; (3) complicated with other malignant tumors; and (4) incomplete medical records.

### Observation Indicators

The clinical information of the patients was assessed. The following variables were statistically analyzed: age at diagnosis, clinical-stage, histology, grading of the tumor, the status of pelvic lymph nodes, surgical margins, parametrial involvement, lymphovascular space invasion, depth of cervical invasion, tumor size, adjuvant therapy after surgery, date of recurrence, and death or last follow-up. It was important to note that the tumor size of patients in our study was evaluated postoperatively by a 5-year experienced gynecologic pathologist.

### Treatment

After radical hysterectomy with pelvic lymphadenectomy, patients with two intermediate prognostic risk factors, such as bulky tumor size, deep cervical stromal invasion, or lymphovascular space invasion, were advised to undergo adjuvant treatment (10). Besides, if the histopathological report revealed at least one of the high-risk prognostic factors, including positive lymph nodes, positive parametrium, or positive surgical margins, adjuvant treatment was needed (17).

### Endpoints

Survival analysis was the primary objective of this study, and recurrence and death were selected as the adverse endpoints. Disease-free survival (DFS) defines as the time elapsed between the date of initial surgery and the first recurrence, which was defined by clinical or imaging evidence and was confirmed pathologically, and the date of the last visit by a patient with no evidence of disease. Overall survival (OS) defines as the period between the month of surgery and death, or the date of the last visit. The follow-up period is the date between surgery to the last follow-up or the time of death.

### Statistical Analysis

**Figure 1:**
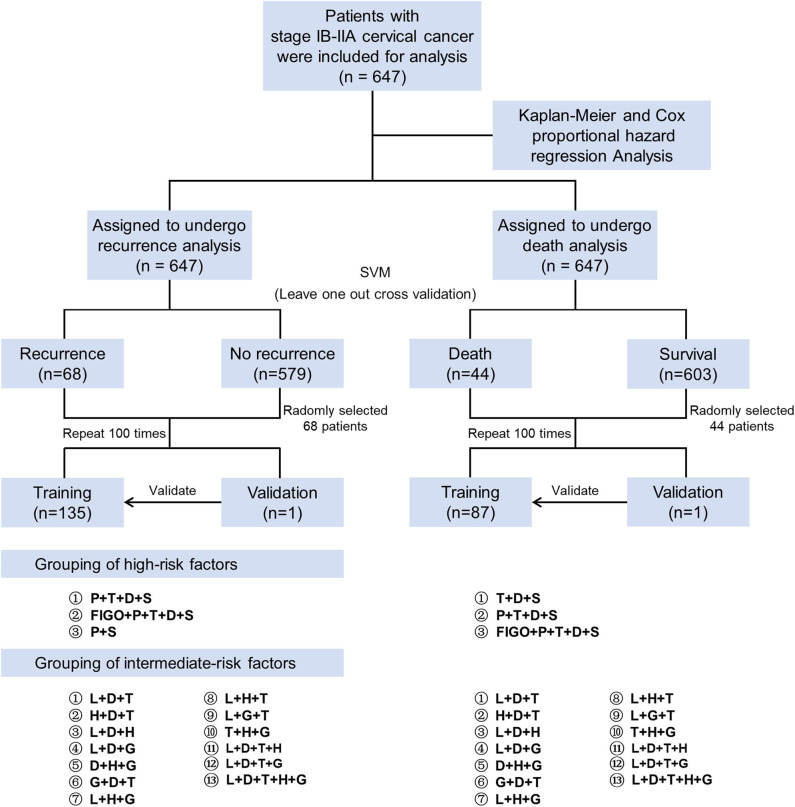
Study flow chart. First, we used the Kaplan-Meier method and Cox proportional hazard regression analysis to screen the high-risk factors for patient prognosis. Then the leave-one-out-cross-validation of the SVM algorithm was used to verify the prognosis prediction accuracy with the different combinations of risk factors. High-risk factors were divided into three groups, and intermediate-risk factors were divided into 13 groups (including the Sedlis criteria). SVM, support vector machine; P, parametrium involvement; T, tumor size; D, deep of cervical stromal invasion; S, status of lymph nodes; FIGO, International Federation of Gynecology and Obstetrics; L, lymphovascular space invasion; H, histology; G, grade.

The study flow chart is presented in Figure 1. Descriptive statistics were used to present the clinical characteristics of patients, and classified data were expressed as numbers and percentages. The Kaplan-Meier method with the log-rank test was selected to perform univariate analysis of DFS and OS. Then, risk factors with a *P* < 0.05 were enrolled in the multivariate Cox proportional hazard regression analysis. The results were described as the hazard ratio (HR), 95% confidence interval (CI), and *P*-value.

We applied the support vector machine (SVM) algorithm to further evaluate the impact of different combinations of risk factors on the prognosis of patients. In the validation process of the SVM algorithm, there were only 68 recurrences in the endpoint cohorts, so we randomly selected 68 cases among the no recurrence cohort to avoid the bias of the two cohorts. Then, we randomly assigned these 136 patients into training (*n* = 135) or validation (*n* = 1) cohort to assess the accuracy of risk factors in predicting adverse endpoints. This process was repeated 100 times. Similarly, we randomly selected 44 cases in the survival cohort and randomly assigned 88 patients to the training (*n* = 87) and validation (*n* = 1) cohorts.

The SVM algorithm is a common classification method in machine learning that accomplishes the task of classification and recognition by constructing a hyperplane. Based on the characteristics of clinical data, a non-linear SVM based on a Gaussian kernel function was applied in this study. SVM calculated the predicted accuracy of different combinations of high-risk factors for recurrence. These factors include the combination of three independent high-risk factors (status of lymph node, parametrial involvement and depth of cervical invasion), four high-risk factors (status of lymph node, parametrial involvement, tumor size, and depth of cervical invasion), and five high-risk factors (FIGO stage, status of lymph node, parametrial involvement, tumor size, and depth of cervical invasion). Different combinations of high-risk factors for survival were examined, such as two independent high-risk factors (status of lymph node and parametrial involvement), four high-risk factors (status of lymph node, parametrial involvement, tumor size, and depth of cervical invasion), and five high-risk factors (FIGO stage, status of lymph node, parametrial involvement, tumor size, and depth of cervical invasion).

Finally, we combined widely recognized intermediate-risk pathological factors (such as risk factors in Sedlis criteria, histology, and grade) and used the SVM algorithm to assess the predictive accuracy of each group for patient recurrence and death. Then, the obtained accuracy was expressed as the median and range, and compared using the Mann-Whitney U-test between different intermediate-risk factor groups.

The log-rank test and multivariate Cox proportional hazard regression analysis were conducted with R software (version 3.6.1) and a *P* < 0.05 was considered significant. The SVM algorithm and Mann-Whitney U test were conducted with MATLAB (version 2016a).

## Results

### General Patient Characteristics

Table 1 summarizes the characteristics of the 647 patients with cervical cancer. The median age of the patients was 45 years (range 21 to 79 years). Preoperative staging of the 647 patients by the FIGO 2009 criteria showed stage IB1 in 431 (66.6%) patients, stage IB2 in 150 (23.2%) patients, stage IIA1 in 37 (5.7%) patients, and stage IIA2 in 29 (4.5%) patients. Lymphovascular space invasion was observed in 179 (27.7%) patients, and pelvic lymph node metastasis was discovered in 153 (23.6%) patients. After a median follow-up period of 29 months (range 6–145 months), 68 recurrences and 44 deaths were identified.

**Table 1:** Clinical characteristics and risk factors related to DFS and OS in the long-rank test.

**Characteristic**	**Total (*n =* 647)**	**DFS**			**OS**		
		**No (*n =* 579)**	**Yes (*n =* 68)**	***P-value***	**No (*n =* 603)**	**Yes (*n =* 44)**	***P-value***
**Age, years**				0.147			0.233
≤ 40	180 (27.8)	165 (28.5)	15 (22.1)		170 (28.2)	10 (22.7)	
>40	467 (72.2)	414 (71.5)	53 (77.9)		433 (71.8)	34 (77.3)	
**FIGO stage (2009)**				0.049			0.038
IB1	431 (66.6)	397 (68.6)	34 (50.0)		410 (48.0)	21 (47.7)	
IB2	150 (23.2)	127 (21.9)	23 (33.8)		135 (22.4)	15 (34.1)	
IIA1	37 (5.7)	31 (5.4)	6 (8.8)		32 (5.3)	5 (11.4)	
IIA2	29 (4.5)	24 (4.1)	5 (7.4)		26 (4.3)	3 (6.8)	
**Grade**				0.494			0.902
I (well)	49 (7.6)	46 (8.0)	3 (4.5)		45 (7.5)	4 (9.1)	
II (moderately)	194 (30.0)	175 (30.2)	19 (27.9)		181 (30.0)	13 (29.5)	
III (poorly)	404 (62.4)	358 (61.8)	46 (67.6)		377 (62.5)	27 (61.4)	
**Histology**				0.244			0.735
Squamous	531 (82.2)	480 (82.9)	52 (76.5)		497 (82.4)	35 (79.7)	
Non-squamous	115 (17.8)	99 (17.1)	16 (23.5)		106 (17.6)	9 (20.5)	
**Status of resection margins**				0.111			0.421
Positive	7 (1.1)	5 (0.9)	2 (2.9)		6 (1.0)	1 (2.3)	
Negative	640 (98.9)	574 (99.1)	66 (97.1)		597 (99.0)	43 (97.7)	
**Parametrium involvement**				0.002			0.001
Yes	17 (2.6)	12 (2.1)	5 (7.4)		13 (2.2)	4 (9.1)	
No	630 (97.4)	567 (97.9)	63 (92.6)		590 (97.8)	40 (90.9)	
**Lymphovascular space invasion**				0.728			0.458
Yes	179 (27.7)	163 (28.2)	16 (23.5)		169 (28.0)	10 (22.7)	
No	468 (72.3)	416 (71.8)	52 (76.5)		434 (72.0)	34 (77.3)	
**Tumor size**				0.028			0.037
≤ 4cm	538 (84.7)	497 (85.8)	51 (75.0)		516 (85.6)	32 (72.7)	
>4cm	99 (15.3)	82 (14.2)	17 (25.0)		87 (14.4)	12 (27.3)	
**Depth of cervical stromal invasion**				0.001			0.006
<1/3	153 (23.6)	145 (25.0)	8 (11.8)		148 (24.6)	5 (11.4)	
1/3~2/3	221 (34.2)	206 (35.6)	15 (22.0)		213 (35.3)	8 (18.2)	
>2/3	273 (42.2)	228 (39.4)	45 (66.2)		242 (40.1)	31 (70.4)	
**Status of lymph node**				<0.001			<0.001
Positive	153 (23.6)	122 (21.1)	31 (45.6)		130 (21.6)	23 (52.3)	
Negative	494 (76.4)	457 (78.9)	37 (54.4)		473 (78.4)	21 (47.7)	
**Adjuvant therapy**				0.809			0.374
Yes	538 (84.7)	490 (84.6)	58 (85.3)		509 (84.4)	39 (88.6)	
No	99 (15.3)	89 (15.4)	10 (14.7)		94 (15.6)	5 (11.4)	

*Values are n (%). DFS, disease-free survival; OS, overall survival; FIGO, International Federation of Gynecology and Obstetrics*.

### Univariate Kaplan-Meier Analysis

**Figure 2:**
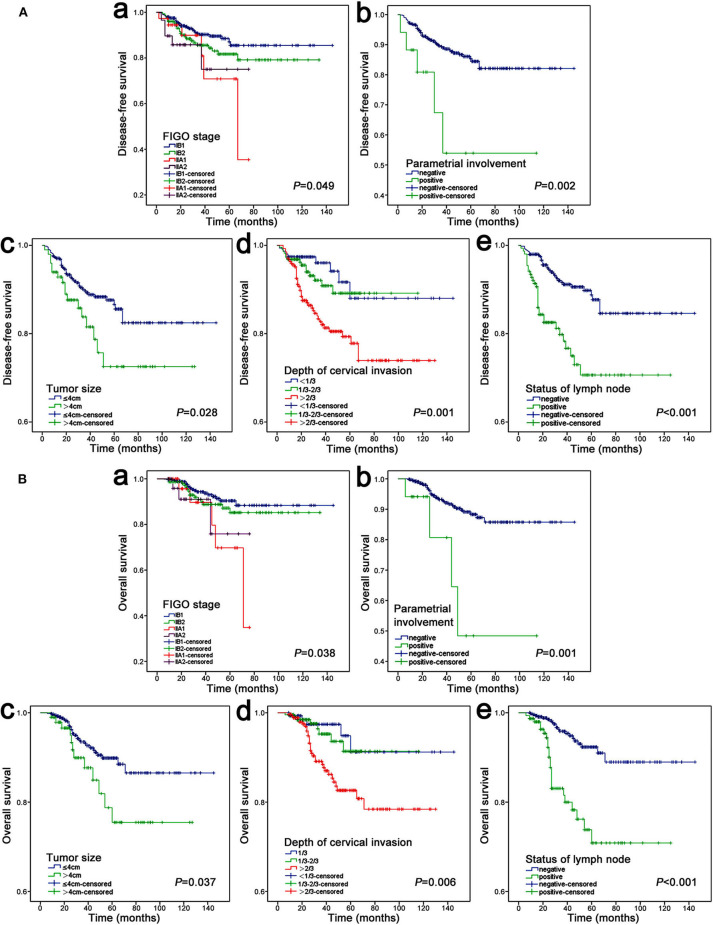
Kaplan–Meier analysis of DFS **(A)** and OS **(B)**. **(A**,a**)** FIGO stage, **(A**,b**)** parametrial involvement, **(A**,c**)** tumor size, **(A**,d**)** depth of cervical invasion, **(A**,e**)** status of lymph node; **(B**,a**)** FIGO stage, **(B**,b**)** parametrial involvement, **(B**,c**)** tumor size, **(B**,d**)** depth of cervical invasion, **(B**,e**)** status of lymph node.

During the univariate analysis, the FIGO stage (2009), parametrial involvement, tumor size, depth of cervical stromal invasion and pelvic lymph node were associated with both recurrence and death (Table 1). Figure 2 shows the Kaplan-Meier curves of the above statistically significant risk factors for DFS and OS. Notably, age, histology, grade and lymphovascular space invasion were not significantly related to recurrence and death in our study (*P* > 0.05). Additionally, we also evaluated the risk of the pathological status of resection margins, only 7 (1.1%) patients had residual lesions at the resection margins after surgery, and no significant association was observed with DFS and OS.

### Multivariate Cox Proportional Hazard Analysis

**Figure 3:**
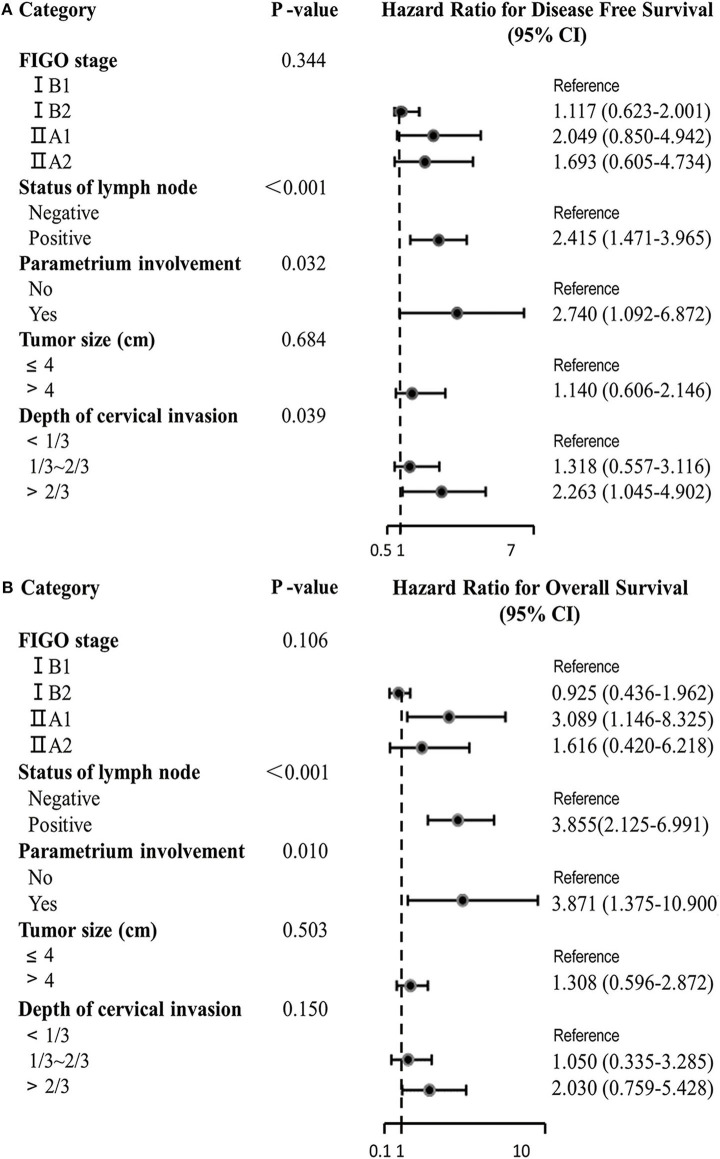
Multivariate Cox proportional hazards regression analysis of DFS **(A)** and OS **(B)**.

In the Cox proportional hazard multivariable analysis, there was a significant correlation between deep of cervical stromal invasion and DFS (*P* = 0.039), especially >2/3 cervical invasion (HR 2.263, 95% CI 1.045–4.902), but it did not seem to have a significant impact on OS (*P* = 0.150). In addition to deep of cervical invasion, lymph node metastasis and parametrial involvement were also found to be independent indicators for DFS (Figure 3A). In addition, lymph node metastasis and parametrial involvement were found to be independent indicators for OS (Figure 3B).

### Validation of the High-Risk Factors Based on the SVM Algorithm

**Figure 4:**
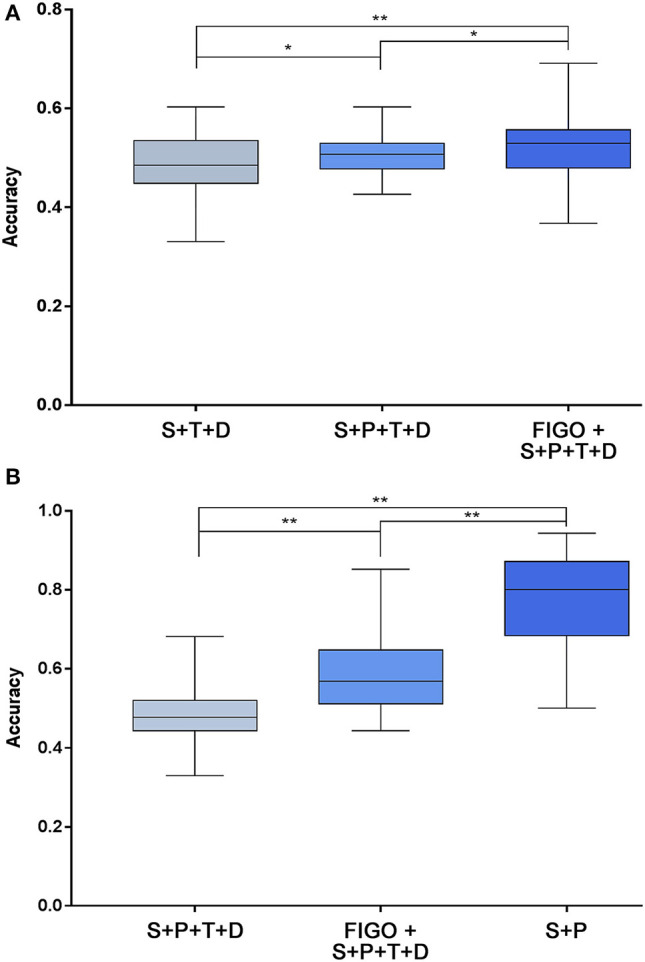
Prediction accuracy of high-risk factors for recurrence **(A)** and death **(B)** based on the SVM algorithm. After combining the screened high-risk factors, the SVM algorithm was used to predict the recurrence and death of each patient. **(A)** Shows the predictive accuracy of a combination of 3 independent high-risk factors (status of lymph node + parametrium involvement + deep of cervical stromal invasion), 4 high-risk factors (status of lymph node + parametrium involvement + tumor size + deep of cervical stromal invasion), and 5 high-risk factors (FIGO stage + status of lymph node + parametrium involvement + tumor size + deep of cervical stromal invasion) on adverse endpoints of recurrence. There are significant differences in accuracy between the three groups (*P* < 0.05). **(B)** Shows the predictive accuracy of a combination of two independent high-risk factors (status of lymph node + parametrium involvement), 4 high-risk factors, and 5 high-risk factors (same as **A**) on adverse endpoints of death. There were also significant differences in accuracy between the three groups (*P* < 0.001). *P < 0.01 and **P < 0.001. SVM, support vector machine; P, parametrium involvement; T, tumor size; D, deep of cervical stromal invasion; S, status of lymph nodes; FIGO, International Federation of Gynecology and Obstetrics.

The prediction accuracy of multiple high-risk factors for recurrence and death is shown in Figure 4. For the prediction of recurrence, the prediction accuracy of the three independent high-risk factors ranged from 33.1 to 60.3%, four high-risk factors ranged from 42.6 to 60.3%, and five high-risk factors ranged from 36.8 to 69.1% (Figure 4A). The combination of five high-risk factors, including FIGO staging, was more accurate in predicting recurrence after surgery in patients with early-stage cervical cancer than the other two combinations (*P* < 0.05). For the prediction of death, the prediction accuracy of the two independent high-risk factors ranged from 50.0 to 94.3%, four high-risk factors ranged from 33.0 to 68.2%, and five high-risk factors ranged from 44.3 to 85.2%. The results here are different from those of recurrence. Regarding the accuracy of predicting death, the accuracy of two independent high-risk predictors was higher than that of 4 and 5 high-risk factors (*P* < 0.05) (Figure 4B).

### Validation of the Intermediate-Risk Factors Based on the SVM Algorithm

**Figure 5:**
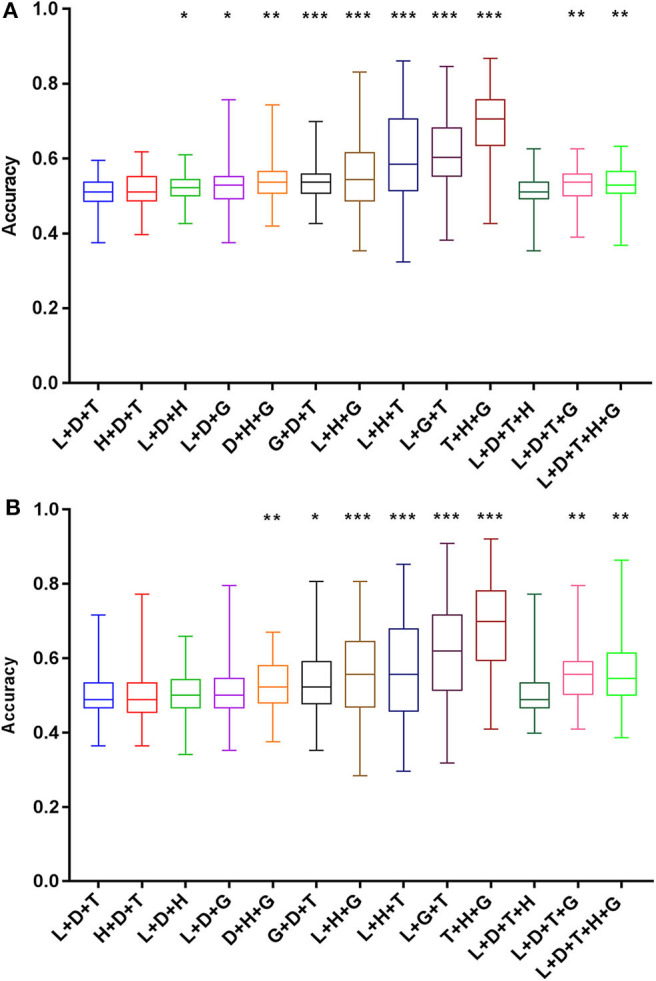
Prediction accuracy of intermediate-risk factors for recurrence **(A)** and death **(B)** based on the SVM algorithm. Histology and grade were recombined with the intermediate-risk factors in the Sedlis criteria, and a total of 13 groups were obtained. The prediction accuracy of each group was compared with that of the L+T+D group. *P < 0.05, **P < 0.01, and ***P < 0.001. SVM, support vector machine; L, lymphovascular space invasion; T, tumor size; D, deep of cervical stromal invasion; H, histology; G, grade.

The prediction accuracy of recurrence by the combination of intermediate-risk factors in the Sedlis criteria (lymphovascular space invasion + tumor size + deep of cervical stromal invasion) ranged from 37.5 to 60.0%. As shown in Figure 5A, among all the combinations, 10 of the intermediate-risk factor groups predicted recurrence accuracy that was significantly higher than that of the Sedlis group (lymphovascular space invasion + tumor size + deep of cervical stromal invasion) (*P* < 0.05). The highest prediction accuracy was obtained in the tumor size, histology, and degree of differentiation group, which ranged from 42.6 to 86.8%. The accuracy in predicting patient death, the group of intermediate-risk factors in the Sedlis criteria, ranged from 36.4 to 71.6%. Eight groups had better predictive accuracy than the group of the Sedlis criteria (*P* < 0.05). The best prediction group included tumor size, histology, and degree of differentiation, and its prediction accuracy ranged from 40.9 to 92.0% (Figure 5B).

## Discussion

This study recombined FIGO staging and surgical-pathological factors of patients with early-staging cervical cancer to explore its accuracy in predicting adverse outcomes after surgery. Risk factors, including FIGO stage, lymph node status, parametrial involvement, tumor size, and depth of cervical cancer, showed significant effects on the prognosis of patients. The SVM-based validation showed that the best prediction accuracy of recurrence was achieved (accuracy = 69.1%) in the combination of the above five high-risk factors. Moreover, the highest survival prediction accuracy was 94.3% when two independent predictors (pathological status of lymph node and parametrium involvement) were combined. In addition, we regrouped intermediate-risk factors. The combination of tumor size, histology and grade of differentiation was more accurate in predicting prognosis than the intermediate-risk factors in the Sedlis criteria.

The FIGO staging system is a widely accepted staging method for cervical cancer in developing countries where modern imaging modalities are not widely available (18). Until the promulgation of the 2014 FIGO staging system, the staging of cervical cancer is mainly based on the characteristics of the primary tumor (12). In 2018, the new FIGO staging system of cervical cancer defined patients with lymph node metastasis as stage IIIC (15). This also illustrates the influence of positive lymph nodes on the prognosis of cervical cancer patients, and the surgical–pathological risk factors will gradually enter the staging of cervical cancer. In our study, FIGO staging was the major high-risk factor associated with both OS and DFS in the univariate log-rank analysis. Nevertheless, the results of the multivariable analysis showed that FIGO staging is not an independent risk factor for prognosis. At the same time, with the analysis of the SVM algorithm, five high-risk factors, including FIGO staging, were more accurate in predicting recurrence of patients than the other two groups. The accuracy of FIGO staging in death prediction is lower than the combination of two pathological factors (status of lymph node and parametrium involvement). Therefore, our study suggested that the FIGO staging system plays an essential role in predicting recurrence when it is combined with other pathological risk factors, but its value cannot be translated into survival benefits.

Several studies have tried to identify prognostic factors in cervical cancer. In studies by Lai et al. and Kamura et al., parametrium involvement was observed in the survival of patients (19, 20). In the analysis of tumor size, we grouped the tumor size using a limit of 4 cm, and the diameter of the tumor was the result of the postoperative pathology, which is different from previous studies (21, 22). Pelvic lymph node metastasis has been observed in several studies and is included in the latest FIGO staging (15, 23, 24). It is also critical to evaluate the condition of pelvic lymph nodes before surgery (23, 25).

Our study showed that in patients with FIGO stage IB-IIA, there is no difference in the prognosis between minimally invasive surgery and abdominal surgery. The same result was obtained in the study of Corrado et al., but the difference is that in their study, all patients were diagnosed with FIGO stage IB1 (26). Two retrospective studies have shown that patients with early-stage cervical cancer treated with minimally invasive surgery had shorter survival times than those undergoing abdominal surgery, but there was no significant difference in the subgroup of patients with tumor diameter <2 cm (27, 28) Anchora et al. showed that in early-stage cervical cancer, patients with >2 cm disease should undergo abdominal surgery, and for patients with tumor <2 cm, both approaches appear safe (29). Patients with early-stage cervical cancer should be provided with more personalized and tailored treatment to improve clinical prognosis.

Compared with the previous study of prognosis assessment of patients with early cervical cancer, our study focused on the analysis of surgical-pathological risk factors and verified the prediction accuracy based on the SVM algorithm. Good prognostic accuracy was achieved during SVM-based validation. In addition, we also assessed the intermediate pathological risk factors including Sedlis criteria that were widely considered. According to the Sedlis criteria, the intermediate-risk group is defined by including various combinations of the three factors (lymphovascular space invasion, depth of cervical cancer, and tumor size), although because of their complexity, half of the recurrences occurred in patients who did not meet the Sedlis criteria (5). Our present study demonstrated that the accuracy of the survival and recurrence prediction of intermediate-risk factors by the Sedlis criteria ranged from 36.4 to 71.6% and 37.5 to 60.0%, respectively. To identify whether there is a better combination of other risk factors to predict recurrence and survival for patients with cervical cancer, we tried to replace the intermediate-risk factors in the Sedlis criteria with other surgical–pathological risk factors, excluding high-risk factors. Our results indicated that the model of tumor size, histology, and grade of differentiation was a better predictor of recurrence and survival than the model of intermediate-risk factors in the Sedlis criteria (42.6 to 86.8% vs. 37.5 to 60.0%; 40.9 to 92.0% vs. 36.4 to 71.6%). We considered that this difference in performance arises from the inclusion of histologic cell type and degree of differentiation as intermediate-risk factors, which were ignored in the traditional intermediate-risk factors in the Sedlis criteria (30).

It is generally believed that adenocarcinomas metastasize early, resulting in a worse prognosis than squamous carcinoma (31, 32). Park et al. investigated patients with stage IA2-IIA cervical cancer and found that non-squamous histology was an independent indicator of DFS and OS (6). Nakanishi et al. had previously demonstrated that the prognosis of patients with adenocarcinoma was poorer than that of patients with squamous cell carcinoma in the presence of lymph node metastasis (33). In addition, the grade of the tumor was shown to be an independent factor associated with both OS and DFS in previous studies (5, 13). However, the histologic type of tumor and the grade of the tumor are not listed in the Sedlis criteria. Our results demonstrated that including histologic cell type and degree of differentiation in the model could dramatically improve the accuracy of criteria for predicting recurrence and survival among patients with early cervical cancer.

Our study evaluated the prognosis of early-stage cervical cancer by using the SVM algorithm, which offers superior prediction performance in both linear and non-linear problems (34). Based on the characteristics of the risk factors, we employed non-linear SVM to train and validate each sample (35). An additional sample was used to evaluate the statistical accuracy of the SVM. Each selected patient was validated using a leave-one-out-cross-validation, and this was considered a good validation alternative when no independent test set was available (36). We obtained the prediction accuracy of prognosis in patients with cervical cancer among different combinations with high-risk factors, and compared the accuracy between different groups. SVM algorithm further validated the high-risk factors obtained and makes our research more complete.

Owing to the limitation of retrospective analysis, our study may have biases in the process of patient selection, and we did not adopt the latest cervical cancer FIGO staging system. In addition, many patients with stage IA2 cervical cancer did not undergo radical hysterectomy with pelvic lymphadenectomy, and these patients were not included in the final study. Finally, for the assessment of intermediate-risk factors, we did not carry out a detailed grouping of tumor sizes. The latest FIGO staging criteria and the more precise Sedlis criteria evaluation will be included as part of our next study.

## Conclusion

This study carried out a comprehensive analysis of the FIGO staging system and surgical-pathological risk factors in patients with early-stage cervical cancer. Overall, the combination of clinical-stage and pathological factors can further enhance the prediction accuracy of the prognosis. In addition, the combination of tumor size, histology and grade of differentiation was more accurate in predicting prognosis than the Sedlis criteria. Our results may favor the development of the decision-making system after surgery therapy and have potential clinical value in the precise treatment of cervical cancer.

## Data Availability Statement

The original contributions presented in the study are included in the article/supplementary files, further inquiries can be directed to the corresponding author/s.

## Ethics Statement

This study was approved by The Ethical Committee of Qilu Hospital of Shandong University (2018066) and waived the need for written, informed consent.

## Author Contributions

LX and RC: data collection and analysis, writing-initial draft, and accomplishing the final version. SY, ZW, and XS: data analysis. KW, XZ, JL, ZZ, TD, XY, KS, and BK: surgeons of the patients. KS and XQ: study concept, design, supervision, and revision of the article. All authors contributed to the article and approved the submitted version.

## Conflict of Interest

The authors declare that the research was conducted in the absence of any commercial or financial relationships that could be construed as a potential conflict of interest.
